# Combinatorial PD-1 Blockade and CD137 Activation Has Therapeutic Efficacy in Murine Cancer Models and Synergizes with Cisplatin

**DOI:** 10.1371/journal.pone.0084927

**Published:** 2013-12-19

**Authors:** Huafeng Wei, Likun Zhao, Wei Li, Kexing Fan, Weizhu Qian, Sheng Hou, Hao Wang, Min Dai, Ingegerd Hellstrom, Karl Erik Hellstrom, Yajun Guo

**Affiliations:** 1 International Joint Cancer Institute, Second Military Medical University, Shanghai, China; 2 School of Bioscience and Bioengneering, South China University of Technology, Guangzhou, China; 3 School of Pharmacy, Liaocheng University, Liaocheng, China; 4 Department of Pathology, Harborview Medical Center, University of Washington, Seattle, Washington, United States of America; Leiden University Medical Center, Netherlands

## Abstract

There is an urgent need for improved therapy for advanced ovarian carcinoma, which may be met by administering immune-modulatory monoclonal antibodies (mAbs) to generate a tumor-destructive immune response. Using the ID8 mouse ovarian cancer model, we investigated the therapeutic efficacy of various mAb combinations in mice with intraperitoneal (i.p.) tumor established by transplanting 3 × 10^6^ ID8 cells 10 days previously. While most of the tested mAbs were ineffective when given individually or together, the data confirm our previous finding that 2 i.p. injections of a combination of anti-CD137 with anti-PD-1 mAbs doubles overall survival. Mice treated with this mAb combination have a significantly increased frequency and total number of CD8^+^ T cells both in the peritoneal lavage and spleens, and these cells are functional as demonstrated by antigen-specific cytolytic activity and IFN-γ production. While administration of anti-CD137 mAb as a single agent similarly increases CD8^+^ T cells, these have no functional activity, which may be attributed to up-regulation of co-inhibitory PD-1 and TIM-3 molecules induced by CD137. Addition of the anti-cancer drug cisplatin to the 2 mAb combination increased overall survival >90 days (and was probably curative) by a mechanism which included a systemic CD8^+^ T cell response with tumor specificity and immunological memory. Strikingly, combined treatment of cisplatin and CD137/PD-1 mAb also gave rise to the long-term survival of mice with established TC1 lung tumors. A similar combination of the 2 mAbs and cisplatin should be considered for clinical ‘translation’.

## Introduction

Epithelial ovarian carcinoma (EOC) is the leading cause of death from gynecologic malignancies in the United States and is the fourth most common cause of cancer death in women [1]. Over 70% of women with EOC present with advanced stage disease and tumor dissemination throughout the peritoneal cavity [2]. The standard treatment for ovarian cancer is surgical debulking followed by platinum-taxane based chemotherapy [3]. Cisplatin and its platinum derivatives are first-line chemotherapeutic agents in the treatment of ovarian cancer. Cisplatin induces apoptosis by irreversibly intercalating DNA through inter- and intrastrand DNA adducts, thereby inducing DNA damage and activation of the apoptotic machinery [4]. Most patients are responsive to chemotherapy at first; however, the majority will eventually have a relapse and die of the disease. Therefore, novel complementary strategies are needed to improve the outcome of ovarian cancer.

There are several reasons to expect that immunotherapy for EOC could be effective [5]. EOC cells express tumor-associated antigens against which specific immune responses have been detected [6-10]. Studies pioneered by Coukos indicate that immunological mechanisms play an important role in the clinical outcome since there is a close correlation between survival and tumor infiltration with CD3^+^ T cells [11]. EOC metastases are frequently restricted to the peritoneal cavity, which facilitates the local delivery of therapeutic agents [12]. Most patients with advanced disease can be brought into temporary clinical remission where the tumor load is small and therefore more likely to respond [9]. However, clinical success with immunotherapies for EOC has been modest [13]. 

Several recent studies have demonstrated therapeutic efficacy both in mouse models and human patients by administration of mAbs that can modify the immune response when used alone or in combinations. For example, mAbs to CTLA4 have antitumor efficacy with prolonged overall survival in patients with metastatic melanoma, and an anti-CTLA4 mAb is clinically approved by the FDA [14]. Beneficial therapeutic effects have been demonstrated in mice with established tumors [14,15] by engaging CD137 (a.k.a. 4-1BB), using agonist antibodies, dimeric RNA aptamers or tumor cells expressing a surface-attached anti-CD137 single chain antibody [15,16], and the preclinical data have led to clinical trials with humanized mAbs directed against CD137 [17]. Programmed Death 1 (PD-1) protein is a co-inhibitory receptor on T cells with a structure similar to that of CTLA-4 but with a distinct biologic function and ligand specificity [18]. Blockade of the interaction between PD-1 and its ligand, PD-L1, potentiates T-cell immune responses in vitro and mediates antitumor activity [19-21]. The preclinical findings have led to recently reported clinical trials showing that anti-PD-1 and anti-PD-L1 mAbs produce an impressing antitumor activity in non-small cell lung cancer, melanoma and renal-cell cancer with complete regression achieved in some patients [22-24]. 

In spite of the promising antitumor efficacy of several mAbs, many tumors are refractory to treatment with single anti-CD137, anti-PD-1 or anti-CTLA4 mAbs [25,26] and combinations of two or more mAbs may be needed. We recently demonstrated in all of 4 mouse tumor models, including the ID8 clone of the MOSEC murine ovarian cancer, that repeated delivery to the tumor site of a combination of mAbs to CD137/PD-1/CTLA4 caused long-term tumor regressions and even cures and that a mAb combination which also comprised a mAb to CD19 was even more effective [27]. While these data are important by demonstrating that a shift from a Th2 type inflammatory response, which is prevalent in tumors [28-30], to a Th1 type response can be curative, repeated delivery of 3-4 mAbs to tumor sites is not practical for clinical ‘translation’. 

The problem associated with the need for local delivery can be overcome for ovarian cancers since they grow and primarily metastasize in the peritoneal cavity and are thus accessible. Furthermore, the number of needed mAbs may be reduced to two, since we already found that a combination of anti-CD137 and anti-PD-1 mAbs can double survival of mice with established ID8 tumors although it is not curative [28]. Based on these findings we have now compared the *in vivo* therapeutic efficacy, measured as prolonged overall survival, of anti-CD137/PD-1 combination with that of the mAbs given as single agents as well as with other mAbs and mAb combinations. We further investigated the systemic and local immunological mechanisms engaged by single or combined anti-CD137/PD-1 mAbs. Importantly, we subsequently showed that a combination of anti-CD137/PD-1 mAbs with the anti-cancer drug cisplatin significantly prolongs life and is probably curative to 80% of mice with established ID8 carcinoma by a mechanism that involves functional CD8^+^ T cells and has tumor antigen specificity as well as immunological memory. A similar regimen also resulted in long-term survival of 33.3% of mice with established TC1 lung carcinoma. A similar approach should be ‘translatable’ to the clinic. 

## Materials and Methods

### Mice and cell lines

For experiments conducted in China, 6 to 8 -week female C57BL were purchased from the Animal Experimental Center of the Second Military Medical University and animal protocols were approved by the Institutional Review Board of Second Military Medical University. Animal experiments performed in Seattle utilized mice purchased from Charles River Laboratories (Wilmington, MA) and the protocols were approved by the Institutional Review Board of University of Washington.

ID8 is a clone of the MOSEC ovarian carcinoma of C57BL/6 origin [31] and TC1 is a clone derived from primary lung epithelial cells of C57BL/6 mice co-transformed with HPV-16 E6 and E7 [27]. The EL4 murine T cell lymphoma cells are of C57BL/6 origin [32]. ID8 and TC1 tumor cells were cultured in the complete DMEM medium supplemented with 10% FBS (Thermo Scientific, Rockford, IL), 100 U/mL penicillin and 100 μg/mL streptomycin before cell suspensions were prepared and transplanted to mice. The EL4 cells and splenocytes were maintained in a complete medium of RPMI-1640 supplemented with 10% FBS, 25 mM HEPES, 2 mM glutamine, 100 U/mL penicillin and 100 μg/mL streptomycin.

### Reagents

The following monoclonal antibodies (mAb) used in animal experiments were purchased from BioXcell (West Lebanon, NH): anti-CD137 (Clone lob12.3), anti-PD-1 (Clone RMP1-14), anti-CTLA4 (Clone 9D9), anti-NK1.1 (Clone PK136), anti-CD8 (Clone 2.43), anti-CD4 (Clone GK1.5) and control (Clone 2A3). The anti-CD137 antibody of clone 2A was prepared in our lab as described previously [33]. 

### Animal studies

Mice were injected intraperitoneally (i.p.) with 3 × 10^6^ ID8 cells in 0.1 mL of PBS. On days 10 and 14 after tumor inoculation, mice were injected i.p. with 0.5 mg of each mAb in total 0.5 mL of PBS as shown in the figure legends. In experiments with mAb/cisplatin combined therapy, group of mice (10 mice per group) bearing 10 days established ID8 tumor received two doses of control, anti-PD-1, anti-CD137 or anti-PD-1/CD137 mAb (0.5 mg per dose per mouse) at 4 days interval with or without cisplatin (10mg/kg) coadministration at their first treatment. The mice were weighed every second day and checked daily for swollen bellies as indicative of ascites information and for any evidence of toxicity, such as changed behavior, inability to move, eat or drink. Following institutional guidelines, mice were killed when they developed ascites and had a weight increase > 30%. The survival of each mouse was recorded and overall survival was calculated. For combined therapy experiments in the TC1 tumor model, mice (6 per group) were injected subcutaneously (s.c.) with 5 × 10^5^ TC1 cells in 0.1 mL of PBS. On days 5 and 9 after tumor transplantation, mice were injected intratumorally (i.t.) with mAbs and/or cisplatin using the dose/schedule described above. Three perpendicular diameters of s.c. tumors were measured every second day using a caliper and tumor volumes were calculated according to the formula: 1/2 × (length) × (width)^2^. Mice were sacrificed when they seemed moribund or their s.c. tumors reached 10 mm in diameter. 

For assessing the development of immunological memory, 15 long-term surviving mice receiving cisplatin/anti-PD1/CD137 combined therapy (pooled from 2 independent experiments) or 5 age-matched naïve mice (which served as control) were challenged i.p. or s.c. with 3 × 10^6^ ID8 cells or 1 × 10^6^ syngeneic but antigenically different TC1 cells. Tumor growth in mice was evaluated as described above.

For depletion experiments, an anti-CD4 (0.2 mg/mouse), anti-CD8 (0.2 mg/mouse), anti-NK1.1 (0.1 mg/mouse) or control mAb (0.2 mg/mouse) was injected i.p. 48 and 72 hours prior to treatment and every 3-4 days thereafter for the duration of the experiments. The depletion of appropriate cell subsets was confirmed by flow cytometric analysis (data not shown). 

### Evaluation of immune cell subsets in spleens and peritoneal lavages by flow cytometry

Mice which had been transplanted i.p. with ID8 cells were euthanized 7 and 14 days after they had been injected with the anti-PD1, anti-CD137, anti-PD1/CD137 or control as in the therapy experiments. Single cell suspensions from spleens were prepared as described previously [27]. To obtain peritoneal immune cells, 3 ml PBS was injected into the peritoneal cavity of mice with ID8 tumors immediately after euthanasia, their belly was massaged and the fluid was removed, filtered through a 70 μM cell strainer (BD Biosciences, San Jose, CA), washed and immune cells were isolated by using a mouse lymphocyte isolation buffer (Cedarlane, Burlington, Ontario) following the manufacturer’s instruction.

Single-cell suspensions were washed with FACS staining buffer and incubated with mouse Fc receptor binding inhibitor for 10 minutes before staining with antibodies of CD45 (clone 30-F11), CD3 (clone 145-2C11), CD4 (clone GK1.5), CD8 (clone 53-6.7), CD19 (clone eBio1D3), CD11b (clone M1/70), GR-1 (clone RB6-8C5), TIM-3 (clone 8B.2C12) and PD-1 (clone J43; all from eBioscience, San Diego, CA) for 30 minutes. For intracellular staining of FoxP3 (clone FJK-16s; eBioscience), IFN-γ (clone XMG1.2; eBioscience), and IL-10 (clone JES5-16E3; eBioscience), cells were fixed, permeabilized, and stained following the instruction of Cytofix/Cytoperm kit (BD Bioscience). Flow cytometry was performed using FACSCalibur (BD Biosciences) and the lymphocyte population was selected by gating CD45-positive cells. The data were analyzed using Flow Jo software (Tree Star, Ashland, OR). All flow cytometry experiments were performed at least 3 times.

### Evaluation of antigen-specific immune response

Isolated splenocytes from treated mice were cultured in the presence of 10μg/mL H-2Db-restricted mesothelin-derived peptides (mesothelin amino acid 406-414) or control HPV-E7-derived peptide (HPV-E7 49-57; all from Shanghai Science Peptide Biological Technology Co.Ltd., Shanghai) for 3 days. Subsequently, IFN-γ in the supernatants was detected by Mouse IFN-γ Quantikine ELISA Kit (R&D systems, Minneapolis, MN). The results were analyzed after normalization according to the T cell numbers. 

For CTL assays, effector cells were obtained by coculturing 5 × 10^6^ splenocytes with 5 × 10^5^ UV-irradiated ID8 cells for 4 days. Peptide-pulsed EL4 target cells were generated by adding 10 μg/ml of peptide and incubating for 4 hours. CTL activity was measured using the CytoTox96 Non-Radioactive Cytotoxicity Assay kit (Promega, Madison, WI) following the manufacturer’s instructions. In brief, target cells were incubated with varying numbers of effector cells for about 4 hours, and supernatants were then analyzed for lactate dehydrogenase release. The results are expressed as percent specific lysis, calculated as (Experimental release-Spontaneous release/Total release-Spontaneous release) × 100. In some experiments, effector cells were incubated with anti-CD4 or CD8 antibody (10μg/mL) for 2 hours before CTL assay. 

### ELISA

Mice injected i.p. with 3 × 10^6^ ID8 cells 10 day earlier were injected twice at 4 days interval with 0.5 mg of each mAb in 0.5 mL of PBS. Two weeks after the last mAb injection, peritoneal immune cells (1 × 10^6^/well) harvested from treated mice were stimulated in vitro with 50 ng/ml PMA and 1 μg/ml ionomycin for 6 hours prior to the analysis of IL-2 and IFN-γ production in the supernatants by ELISA according to the manual (R&D systems). The results were analyzed after normalization according to the T cell numbers in total peritoneal immune cells.

### Statistics

Results were expressed as mean ± standard error of mean (M±SEM). All statistical analyses were performed using GraphPad Prism 5. Student’s t test was used to compare the statistical difference between two groups and one-way ANOVA was used to compare three or more groups. Survival rates were analyzed using the Kaplan–Meier method and evaluated with the log-rank test. Differences were accepted as significant at p<0.05.

## Results

### Synergistic antitumor effect of anti-CD137/PD-1 mAbs

**Figure 1:**
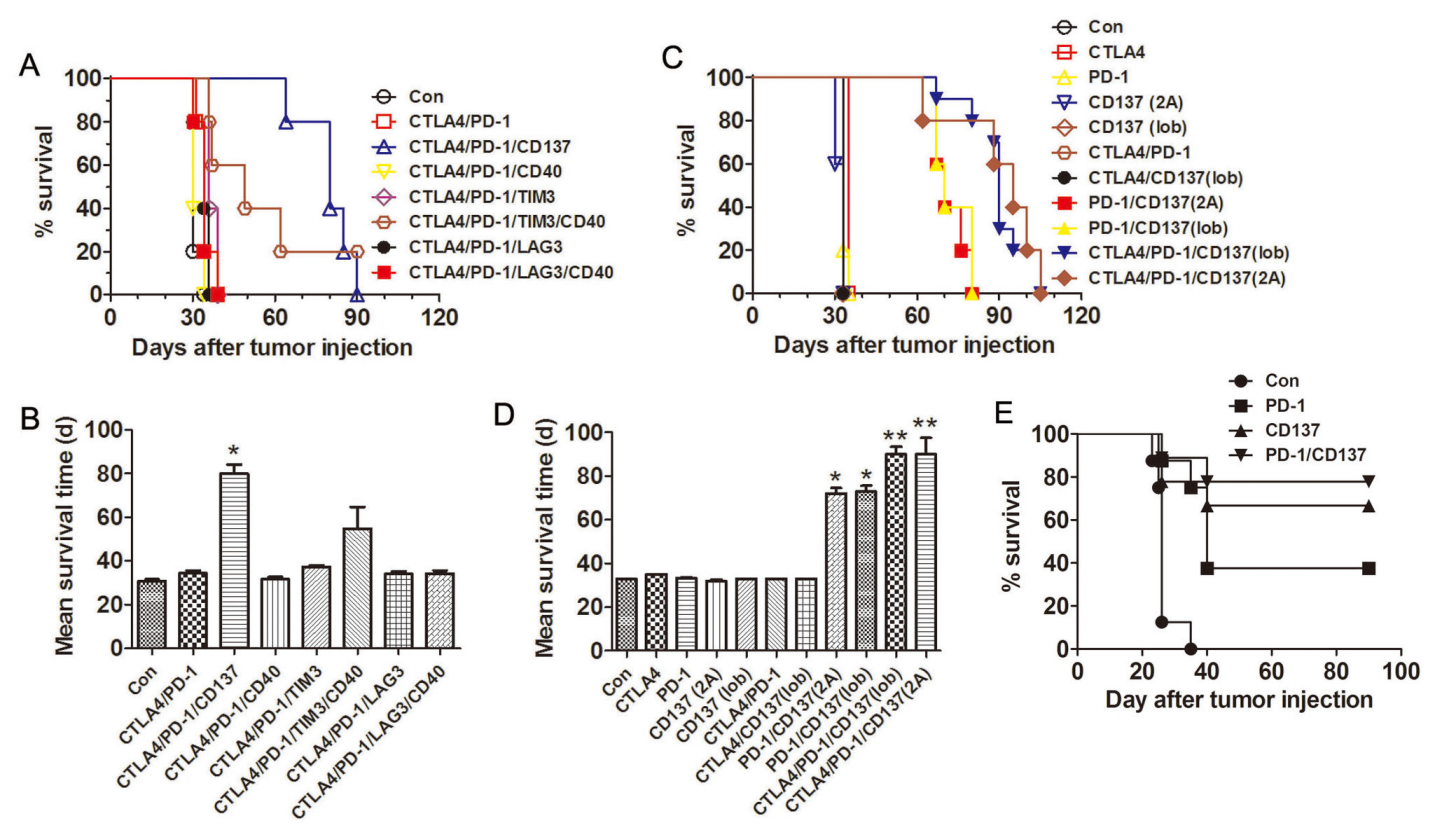
Antitumor effects of anti-CD137/PD-1 mAbs againstne ID8 ovarian cancer. Mice (5/group) transplanted i.p. with 3 × 10^6^ ID8 cells 10 days previously were injected i.p. twice at 4 days interval with the indicated mAb combinations (0.5 mg of each mAb/mouse); survival was recorded (A, C) and mean survival time was calculated (B, D). The experiment was repeated once with similar results. E, Mice (8-9/group) transplanted i.p. with 3 × 10^6^ ID8 cells 3 days previously were injected i.p. twice at 4 days interval with 0.5 mg of control, anti-PD-1, anti-CD137 and anti-PD-1/CD137 mAb and their survival was recorded. *P < 0.05, **P < 0.01, compared with control mAb treated mice.

We evaluated the antitumor efficacy of different combinations of agonistic or antagonistic mAbs against co-stimulatory or co-inhibitory molecules in C57BL mice transplanted i.p. 10 days previously with 3 × 10^6^ ID8 cells. Based on published evidence indicating a therapeutic potentials in several tumor models we tested mAbs to CD137, CD40, CTLA-4, PD-1, TIM-3 and LAG-3 [34,35], as single agents and in combinations. Untreated mice and mice receiving a control mAb developed ascites about 30 days after tumor inoculation and had to be euthanized. As shown in Figure 1, most of the tested mAbs combinations had little or no effects on the overall survival of tumor-bearing mice, and none of the mAbs was therapeutically effective when used alone. Consistent with our recently published results [27], combined treatment of anti-CTLA4, PD-1 and CD137 mAbs significantly prolonged survival of mice with the mean survival time increasing to 80 days (Figure 1A,B; p<0.05 compared to controls). Combined treatment of anti-CTLA4, PD-1, TIM-3 and CD40 mAbs also suppressed tumor growth but was less efficacious with mean survival time 49 days. Other mAb combinations, including anti-CTLA4/PD-1, anti-CTLA4/PD-1/CD40, anti-CTLA4/PD-1/TIM-3, anti-CTLA4/PD-1/LAG-3 and anti-CTLA4/PD-1/LAG-3/CD40 had no effect on tumor growth. 

We next repeated the above experiments by using two different clones of mAb against CD137 (2A and lob12.3). As shown in Figure 1C and D, although single mAb treatment did not prolong overall survival, combination of anti-PD-1 mAb with either anti-CD137 mAb equally prolonged survival of mice with the mean survival time doubled (p<0.05 compared with control and single mAb groups), which was further enhanced by addition of anti-CTLA4 mAb (p<0.01 compared with control and single mAb groups). A repeat of the experiment gave similar results (data not shown). 

Notably, we did not detect any expression of CD137 and PD-1 molecules and their respective ligands CD137L and PD-L1/PD-L2 on the surface of ID8 ovarian cancer cells (data not shown), excluding the possibility that inhibition of ID8 tumor growth *in vivo* is directly mediated by the anti-CD137 plus anti-PD-1 mAbs. 

Interestingly, either anti-CD137 or anti-PD-1 mAb alone protected against outgrowth of ID8 cells in 3 days established ID8 model with respective 66.7% and 37.5% of mice surviving 90 days when the experiment was terminated and the mice euthanized (Figure 1E). The peritoneal cavity of these mice was tumor free. It is noteworthy that anti-CD137 mAb was more effective than anti-PD1 mAb and that a combination of the two was modestly more effective (77.8% surviving mice) than anti-CD137 mAb alone. 

### Increased percentage of effector T cells and decreased percentage of immunosuppressive cells induced by anti-CD137/PD-1 mAbs

**Figure 2:**
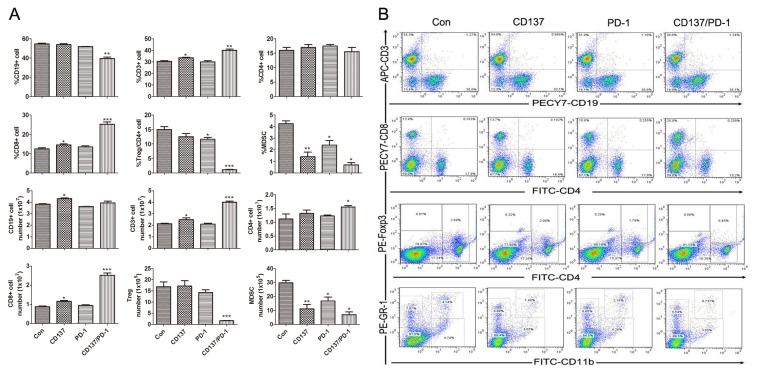
Analysis of lymphocyte components in spleens from mice treated with mAb combinations. Mice (3/group) transplanted i.p. with 3 × 10^6^ ID8 cells 10 days earlier were injected i.p. with 0.5 mg of control, anti-CD137, anti-PD-1 or anti-PD-1/CD137 mAb and the mAb injection was repeated 4 days later. Seven days after the second injection, spleens were harvested and single cell suspensions prepared and stained with fluorescence labeled antibodies against markers of lymphocyte subsets prior to analysis by flow cytometry. The percentages and numbers of CD3^+^, CD4^+^, CD8^+^, CD19^+^, FoxP3^+^/CD4 and GR-1^+^CD11b^+^ cells in spleens are shown in (A) and representative dotplots are shown in (B). Data are presented as M ±SEM from 3 mice/group and are representative of 3 independent experiments. *P < 0.05, **P < 0.01, ***P < 0.001, The findings with anti- PD-1 or CD137 single mAbs are compared with control mAb, and the findings with anti- PD-1/CD137 mAbs are compared with both control and single mAb.

**Figure 3:**
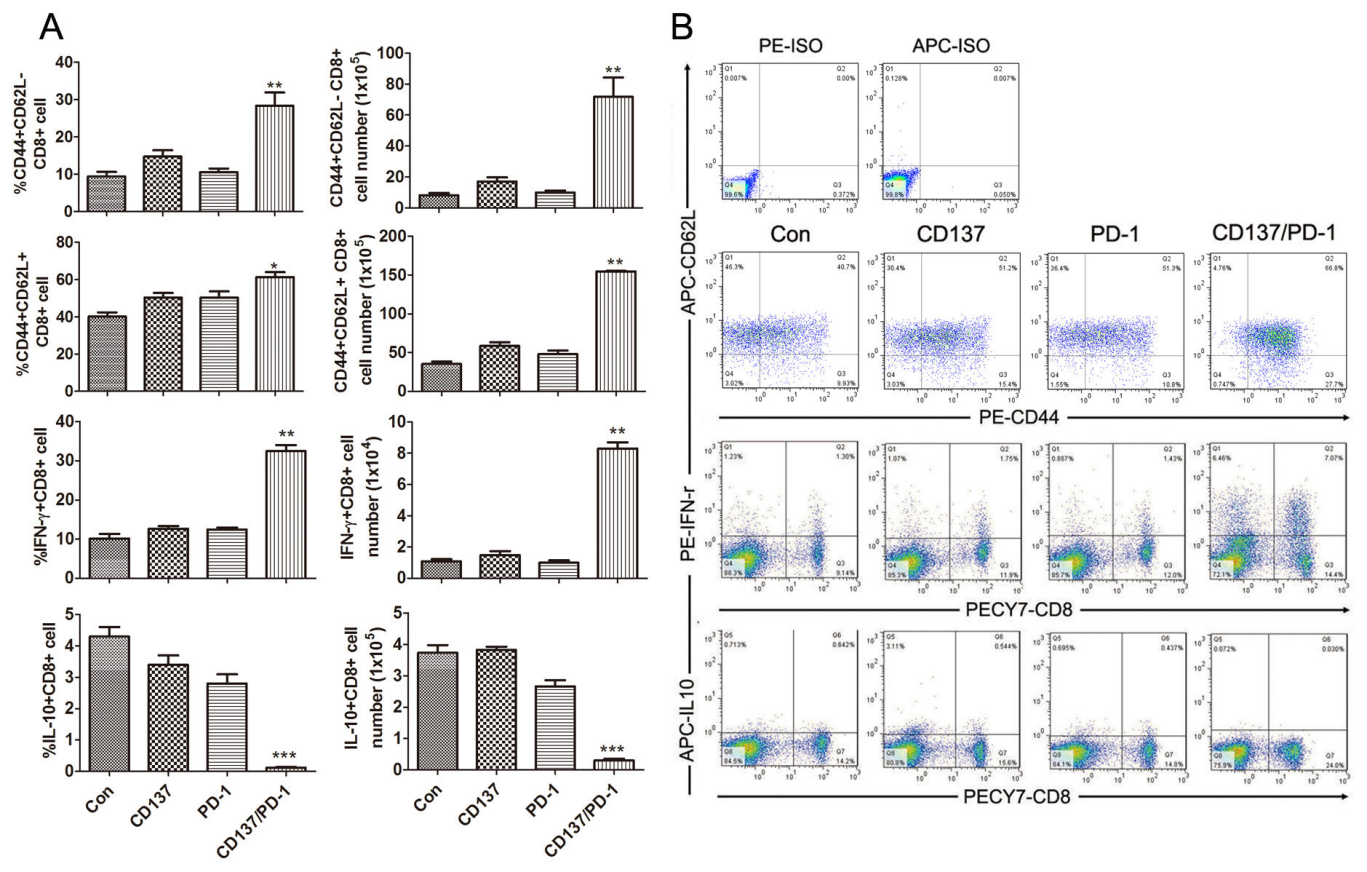
Analysis of effector CD8^+^ T lymphocyte in spleens from mice treated with mAb combinations. Mice (3/group) which had been transplanted i.p. with 3 × 10^6^ ID8 cells 10 days earlier were injected i.p. twice at 4 days interval with 0.5 mg of control, anti-CD137, anti-PD-1 or anti-PD-1/CD137 mAb. Spleens from treated mice were analyzed for phenotypes and effector functions of CD8^+^ T lymphocytes by flow cytometry 7 days after the second mAb injection. The percentages and numbers of CD44^+^CD62L^-^ effector/memory and CD44^+^CD62L^+^ central memory and IFN-γ- and IL-10-producing cells in the CD8^+^ T cell population are shown in (A) and representative dotplots are shown in (B). Data are presented as M±SEM from 3 mice in each group and are representative of 3 independent experiments. *P < 0.05, **P < 0.01, ***P < 0.001, PD-1 or CD137 mAb compared with control mAb, PD-1/CD137 mAb compared with control and single mAb.

To explore whether combined anti-PD-1/CD137 mAbs induced a systemic immune response, we injected mice transplanted 10 days earlier with ID8 cells (as in the therapy experiments) with either a combination of the anti-PD-1/CD137 mAbs or either mAb alone. Seven days later, we analyzed by flow cytometry the percentage, absolute number and effector function of lymphocyte subsets in the spleens from the treated mice. As shown in Figure 2A, spleens from mice treated with combined anti-PD1/CD137 mAbs displayed a significantly increased frequency and absolute number of CD3^+^ (p<0.01) and CD8^+^ T (p<0.001) cells and decreased levels of CD4^+^FoxP3^+^ regulatory T (Treg) cells (p<0.001) and GR-1^+^CD11b^+^ myeloid-derived suppressor cells (MDSCs) (p<0.05); representative plots are shown in (Figure 2B). Combined anti-PD1/CD137 mAbs also elevated the levels of CD44^+^CD62L^-^ effector/memory (p<0.01), CD44^+^CD62L^+^ central memory (p<0.05), IFN-γ-producing effector CD8^+^ T cells (p<0.01) and decreased CD8^+^ T cells producing IL-10 (p<0.001) in the spleens (Figure 3A); representative plots are shown in Figure 3B. The data indicate that anti-PD-1/CD137 mAbs therapy generated a systemic immune response dominated by significantly increased CD8^+^ effector T cells and decreased immunosuppressive cells. 

### Antigen-specific CTL response induced by anti-CD137/PD-1 mAbs

**Figure 4:**
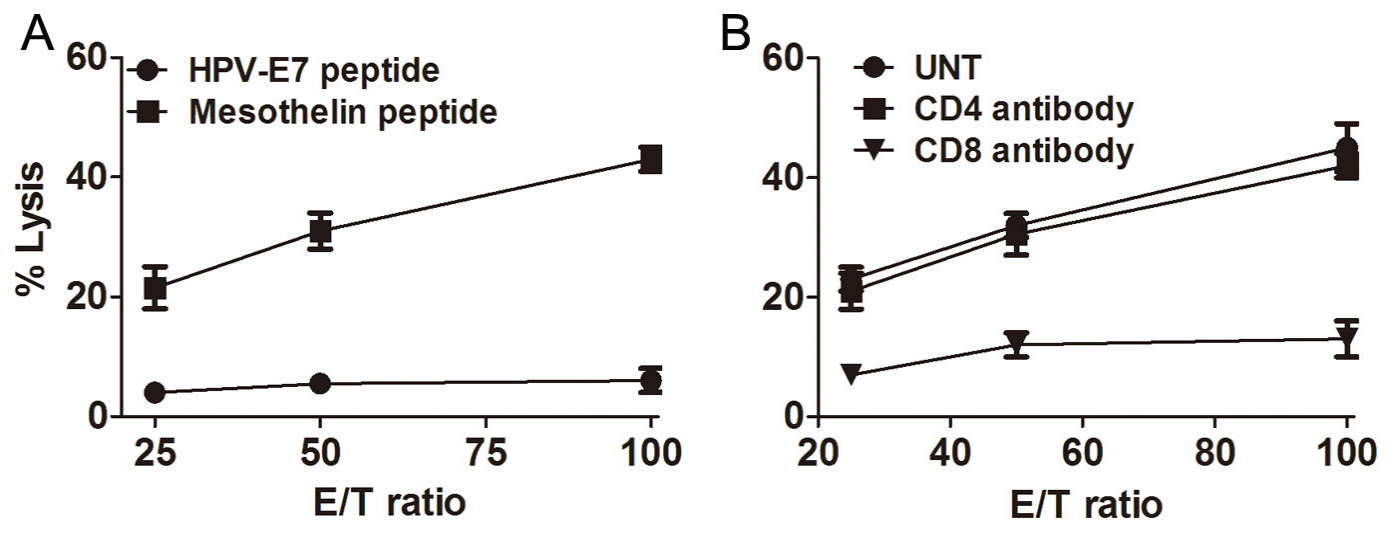
Mice injected with an anti-PD-1/CD137 mAb combination developed a tumor antigen-specific CTL response. Mice (3/group) transplanted i.p. with 3 × 10^6^ ID8 cells 10 day earlier were injected i.p. twice at 4 days interval with 0.5 mg of anti-PD-1/CD137 mAb. Seven days after the second mAb injection, pooled splenocytes (5 × 10^6^) from 3 mice were incubated with 5 × 10^5^ UV-irradiated ID8 cells for 4 days. The resultant splenocytes were then evaluated for antigen-specific CTL activity by CytoTox 96 Non-radioactive cytotoxicity assay using EL4 cells pulsed with H-2Db-restricted mesothelin or HPV-E7 peptide as target cells (A). The killing activity was also evaluated in the presence of anti-CD4, anti-CD8 or control antibody (B). Data were expressed as M±SEM of triplicate wells.

Our previous study demonstrated that combined anti-CTLA4/PD-1/CD137 mAbs induce a potent antigen-specific Th1 type immune response in spleens from ID8-bearing mice [27], whose splenocytes produced high levels of IFN-γ upon stimulation by peptide derived from ID8 cell-expressing mesothelin, a well-characterized ovarian tumor antigen [9]. Similarly, we detected IFN-γ production by splenocytes from mice treated with combined anti-PD-1/CD137 mAbs in response to stimulation by mesothelin but not a HPV-E7 derived peptide used as control (data not shown). We further determined whether the splenocytes had cytolytic activity. Splenocytes from mice treated with anti-PD-1/CD137 mAbs were restimulated with UV-irradiated ID8 cells for 4 days before CTL assays were performed using EL4 cells pulsed with HPV-E7 or mesothelin-derived peptide as target cells. As shown in Figure 4A, splenocytes from anti-PD-1/CD137 treated mice displayed cytotoxic activity on EL4 cell pulsed with mesothelin but not with HPV-E7 peptide. Pre-incubation with CD8 antibody suppressed the killing activity (Figure 4B). We conclude that combined anti-PD-1/CD137 mAbs elicited a tumor antigen-specific CTL response mediated by CD8 cells.

### Local Th1 type immune response promoted by anti-CD137/PD-1 mAbs

**Figure 5:**
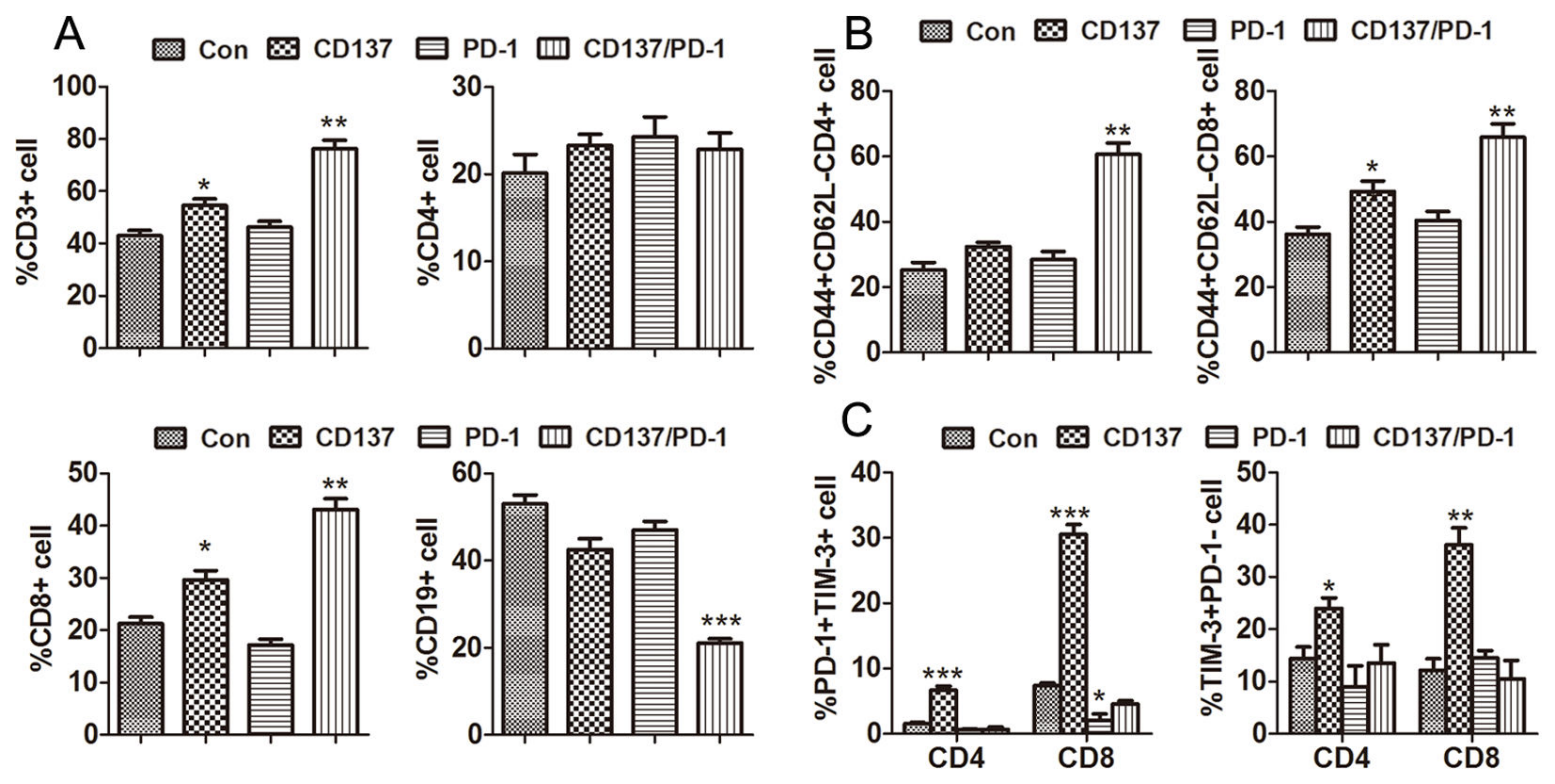
Analysis of peritoneal lymphocyte subsets from mice injected with the anti-PD-1/CD137 combination. Mice (3/group) transplanted i.p. with 3 × 10^6^ ID8 cells 10 day earlier were injected i.p. twice at 4 days interval with 0.5 mg of control, anti-CD137, anti-PD-1 or anti-PD-1/CD137 mAb. Two weeks later, peritoneal lavage from treated mice was analyzed by flow cytometry for the percentage and phenotype of peritoneal lymphocytes. The percentages of CD3^+^, CD4^+^, CD8^+^ and CD19^+^ lymphocytes in peritoneal lavage and CD44^+^CD62L^-^ effector/memory cells in CD4^+^ and CD8^+^ T cells are shown in (A) and (B) respectively. The percentage of PD-1^+^TIM-3^+^ and PD-1^-^TIM-3^+^ cells in peritoneal CD4^+^ and CD8^+^ T cells are shown in (C) with representative dotplots in Figure S2. Data are presented as M±SEM from 3 mice of each group and are representative of 2 independent experiments. *P < 0.05, **P < 0.01, ***P < 0.001, PD-1 or CD137 mAb compared with control mAb, PD-1/CD137 mAb compared with control and single mAb.

To analyze the change in the local immune cells, mice bearing 10-day established ID8 tumor were treated with either single or combined anti-PD-1/CD137 mAbs and their peritoneal lavages were harvested 7 days after the last mAb injection. Confirming our previous findings [27], treatment of anti-PD-1/CD137 mAbs induced significantly increased infiltration of CD3^+^ (p<0.01) and CD8^+^ (p<0.01) cells as well as decreased infiltration of CD19^+^ (p<0.001) cells as compared with either control or single mAb treatment (Figure 5A). Further analysis of CD44 and CD62L expression showed that most of CD4^+^ and CD8^+^ T cells from anti-PD-1/CD137 mAbs treated mice were CD44^+^CD62L^-^ effector/memory T cells and their percentages were much higher (p<0.01) than that from control or single mAb treated mice (Figure 5B); representative dotplots are shown in Figure S1. We also confirmed decreased levels of Tregs and MDSCs as previously reported (data not shown; ref 27).

Single CD137 mAb treated mice also exhibited increased infiltration of CD3^+^ (p<0.05), CD8^+^ (p<0.05) and CD44^+^CD62L^-^ effector/memory CD8^+^ T (p<0.05) cells in the peritoneal cavity although it was ineffective in prolonging overall survival (Figure 1D). To explore the mechanisms underlying the failure of tumor protection induced by anti-CD137 mAb alone, we examined the expression of PD-1 and immune regulator T cell immunoglobulin mucin (TIM-3) co-inhibitory molecules on T cells, which have been reported to be closely associated with functional exhaustion of T cells [36,37]. As shown in Figure 5C, anti-CD137 mAb up-regulated the expression of PD-1 and TIM-3 on peritoneal CD4^+^ and CD8^+^ T cells and single anti-PD-1 mAb had little effects on TIM-3 expression although it attenuated the expression of PD-1 on CD8^+^ T cells compared with the control mAb. Notably, concomitant PD-1 blockade significantly prevented the up-regulation of PD-1 and TIM-3 on T cells induced by CD137 activation; representative dotplots are shown in (Figure S2). Consistent with the upregulation of PD-1 and TIM-3, peritoneal T cells isolated from anti-CD137 mAb treated mice produced lower levels of IL-2 and IFN-γ (Figure S3). 

### Long-lasting antitumor effect induced by combined anti-CD137/PD-1 mAbs and cisplatin

**Figure 6:**
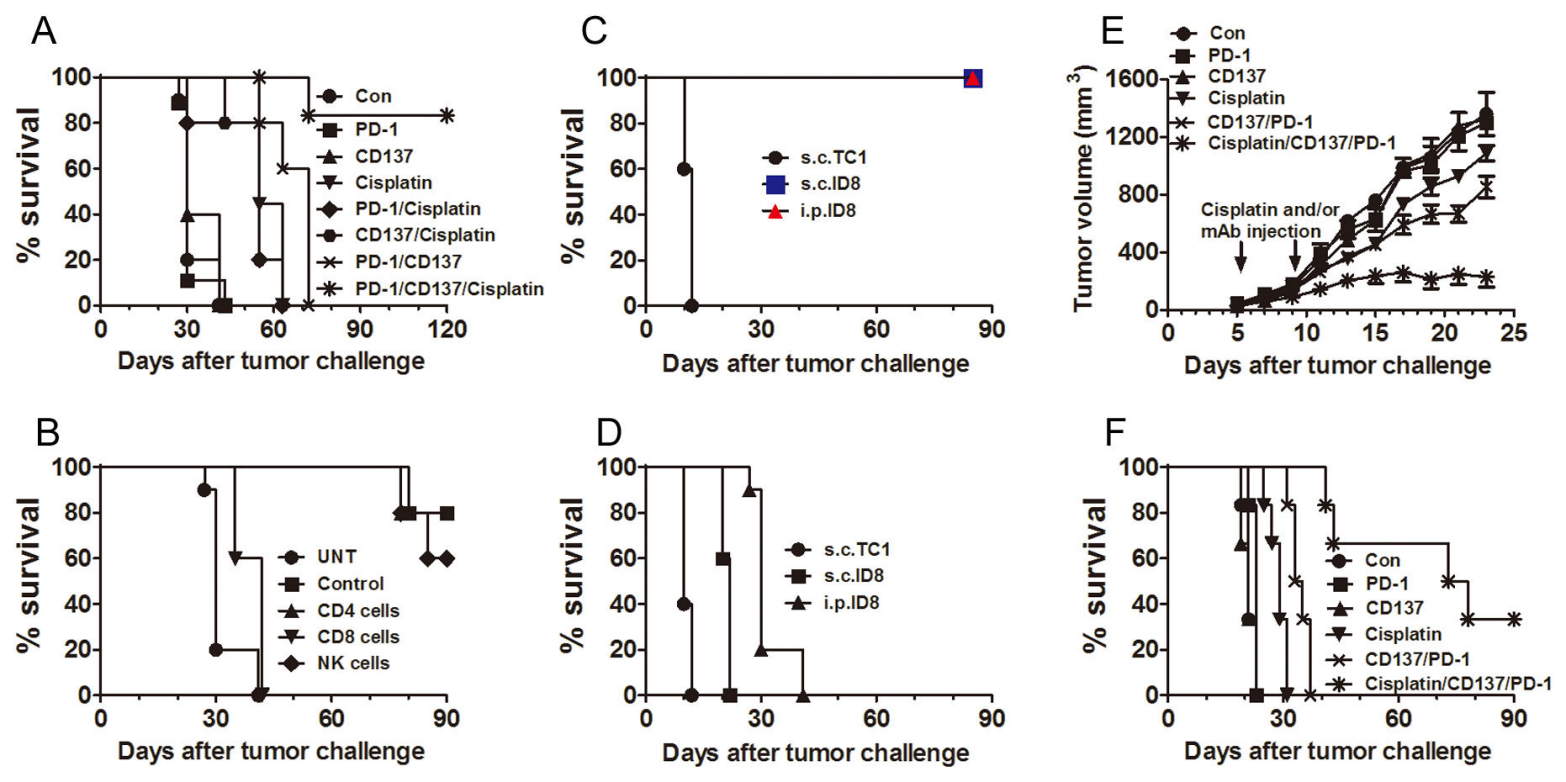
Combining anti-PD-1/CD137 mAb with cisplatin induced complete remission of established ID8 cancer with long-lasting systemic tumor-specific immunity. Mice (10/group) transplanted i.p. with 3 x 10^6^ ID8 cells 10 days earlier were injected i.p. with two doses of control, anti-PD-1, anti-CD137 or anti-PD-1/CD137 mAb (0.5 mg per dose per mouse) at 4 days interval with or without coadministration of cisplatin (10mg/kg) at the first treatment and their survival was evaluated (A). Mice (10/group) treated with combined anti-PD-1/CD137/cisplatin were depleted of lymphocyte subsets by injection of anti-CD4 (0.2 mg/mouse), anti-CD8 (0.2 mg/mouse), anti-NK1.1 (0.1 mg/mouse) or control mAb (0.2 mg/mouse) 48 and 72 hours prior to the first treatment and every 3-4 days thereafter for the duration of the experiments. Untreated tumor-bearing mice were as negative controls (UNT group). The survival of mice was recorded (B). Fifteen long-term surviving mice (120 days after original transplantation of ID8 cells) pooled from 2 experiments were challenged (5 mice/group) with ID8 cells given i.p. or s.c. or with TC1 cells transplanted s.c. (C); naive mice were transplanted with tumor cells as controls (D) and their survival was recorded. Mice (6/group) with established TC1 tumors of 4-5 mm mean diameter were injected i.p. with two doses of control, anti-PD-1, anti-CD137 or anti-PD-1/CD137 mAb (0.5 mg per dose per mouse) at 4 days interval with or without coadministraation of cisplatin (10 mg/kg) at the first treatment; tumor growth was measured (E) and survival was recorded (F). Data are representative of 2 experiments for Figure 6A-D.

Prior studies have reported that anti-CD137 mAb synergizes with cisplatin, a commonly used chemotherapeutic drug for ovarian cancer, to induce regression of the murine CT-26 colon cancer in 60% of mice [38]. To explore the efficacy of anti-PD-1/CD137 mAbs plus cisplatin in the ID8 ovarian cancer model, we first treated tumor-bearing mice with i.p. injection of a dose of cisplatin followed by 2 doses of anti-PD-1/CD137 mAbs at 4 days interval. As shown in Figure 6A, combined anti-PD-1/CD137/cisplatin treatment produced a significant antitumor effect as compared with single mAb, both mAbs or cisplatin alone, resulting in the long-term survival of 80% (8 mice out of 10) mice; there was no survival benefit from cisplatin alone or combined with either anti-PD-1 or anti-CD137 mAbs. We obtained similar results from a repeated experiment. The removal of CD8^+^ T cells completely abrogated the antitumor effect (Figure 6B), demonstrating a pivotal role of these cells in tumor protection. The long-term survivors developed a systemic immune response with memory and tumor-specificity as demonstrated by their resistance to challenge with ID8 cells but not to challenge with antigenically unrelated TC1 cells (Figure 6C). Control, naïve mice succumbed to challenge with either ID8 or TC1 cells which had similar growth rates (Figure 6D). 

To validate the above results, we did an experiment with the TC1 lung tumor model. Mice with s.c. established TC1 tumors (4-5mm in diameter) were injected i.t. with anti-PD-1, anti-CD137, or anti-PD-1/CD137 mAbs, or cisplatin, or a combination of the two mAbs plus cisplatin, using the same dose/schedule as in the ID8 experiment. As shown in Figure 6E and 6F, anti-PD-1 or anti-CD137 mAb had no anti-tumor efficacy as a single agent and cisplatin alone or a combination of anti-PD-1/CD137 mAbs only slightly suppressed tumor growth. In contrast, a combination of anti-PD-1/CD137 with cisplatin significantly inhibited tumor growth (median survival days 21, 21, 23, 29, 34, and 76 days for control, anti-PD-1, anti-CD137, cisplatin, anti-PD-1/CD137 and anti-PD-1/CD137/cisplatin respectively; p<0.05 for anti-PD-1/CD137/cisplatin versus cisplatin or anti-PD-1/CD137) and 2 of 6 mice survived tumor-free when the experiment was terminated 90 days after tumor transplantation.

## Discussion

Antibodies which activate the T-cell co-stimulatory receptor CD137 or block the co-inhibitory receptor PD-1 have demonstrated broad anti-tumor effects but have not improved the survival of mice with poorly immunogenic tumors, including the ID8 murine ovarian cancer, when used as single agents [25,39,40]. In contrast, repeated administration of a combination of mAbs to CD137 plus PD-1 doubles tumor-free survival of mice with established ID8 carcinoma, and addition of a mAb to CTLA4 further increases it [27]. Importantly, a combination of anti-CD137/PD-1/CTLA4 mAbs was shown to be therapeutically efficacious (and sometimes curative) in all of 4 mouse models investigated when delivered to the tumor microenvironment, and the efficacy was further increased by also including a mAb to CD19 [27]. Tumor regression appeared to be caused by a shift from a tumor-promoting Th2 type inflammatory response to a tumor-destructive Th1 response [27]. 

While the published findings provide excellent models to investigate immunological mechanisms associated with tumor regression and cures [28], a protocol based on repeated delivery of 3-4 mAbs to tumor sites is not likely to become part of the clinical mainstay and simplified approaches are needed. Ovarian carcinomas originate and primarily metastasize in the peritoneal cavity, which provides direct access by mAbs injected i.p. for local delivery, a procedure for which there is clinical precedence [41]. However, the need to decrease the number of agents required for long term remissions and the number of doses still remains. This study was performed with that goal in mind, focusing on the administration of a combination of anti-CD137 and anti-PD-1 mAbs to mice with ID8 ovarian carcinoma established i.p. by transplantating 3 × 10^6^ ID8 cells 10 days before treatment. 

 Our first objective was to compare the therapeutic efficacy of a combination of anti-CD137/PD-1 mAbs with that of mAbs to several other immunoregulatory molecules (CD40, TIM-3, LAG-3) given as single agents or in combinations of 2 together. We found that combined anti-CD137/PD-1 mAb was superior to any of the other combinations tested or to single mAbs. 

Our next step was to analyze the mechanisms involved. To evaluate systemic immunity (which is needed for a therapeutic approach to be clinically useful) we harvested spleen cells from treated and control mice and investigated their phenotypes by flow cytometry as well as performed functional assays. Based on published work [26,42], we hypothesized that the anti-CD137 mAb activates and prolongs survival of Th1/Tc1 cells, but, as a side-effect, also increases their expression of the co-inhibitory receptors PD-1 and TIM-3 [36,37], which causes functional exhaustion that can be overcome by administering an anti-PD1 mAb [36]. We found that anti-CD137 mAb, administered as a single agent, promoted the accumulation of effector CD8^+^ T cells in spleens and peritoneal cavity of treated mice; however, these cells produced negligible levels of IFN-γ upon stimulation, suggesting increased CD8^+^ T cells by anti-CD137 mAb either lacked function or was immunologically ignorant [25]. Administration of anti PD-1 mAb as a single agent had little effect on CD4^+^, CD8^+^ T cells and CD19^+^ B cells but significantly decreased the immunosuppressive CD4^+^FoxP3^+^ Treg and CD11b^+^GR-1^+^ MDSC populations in spleens. The therapeutically efficacious combination of anti-PD-1 and anti-CD137 mAbs increased the percentage and absolute number of CD8^+^ effector T cells and decreased levels of immunosuppressive Tregs and MDSCs, giving rise to significantly increased ratios of effector CD8^+^ T cells to immunosuppressive cells. Importantly, these CD8^+^ T cells were functionally active, producing large amounts of IFN-γ upon polyclonal or tumor antigen-specific stimulation and exhibiting antigen-specific cytotoxicity. A pivotal role of CD8^+^ T cells in the ID8 model was further supported by experiments showing that antibody-mediated depletion of CD8^+^ cells abrogated the in vivo therapeutic effect. The combined anti-PD-1/CD137 treatment also increased the absolute number of splenic CD4^+^ T cells and percentage of peritoneal effector CD4^+^ T cells; however, it appeared that CD4^+^ T cells are dispensable for tumor protection in this model since removal of CD4^+^ T cells had little effect on antitumor effect triggered by combined anti-PD-1/CD137 and cisplatin treatment.

Phenotype analysis showed that anti-CD137 mAb induced the upregulation of PD-1 and TIM-3 expression on peritoneal CD4^+^ and CD8^+^ T cells. Recent studies in multiple mouse tumor models have documented the presence of significantly increased PD-1^+^TIM-3^+^ CD8^+^ tumor-infiltrating lymphocytes (TILs) exhibiting an exhausted phenotype as defined by the failure to produce effector cytokines [36,37]. In melanoma patients, up-regulation of TIM-3 and/or PD-1 expression is directly correlated with tumor antigen–specific CD8^+^ T cell dysfunction [37]. Consistent with an exhausted phenotype, freshly isolated peritoneal immune cells from anti-CD137 treated mice produced little IL-2 and IFN-γ upon polyclonal stimulation (Figure S3). Our findings are concordant with a recent study showing that agonist CD137 antibody induced the expression of PD-1 on CD8^+^ and CD4^+^ TILs from mice bearing B16 melanoma when they were immunized with a cell-based vaccine [42]. Consistent with in vivo antitumor effect, combined anti-PD-1/CD137 blocked the upregulation of PD-1 and TIM-3 molecules on peritoneal CD4^+^ and CD8^+^ T cells and reverted their dysfunction. 

Importantly, addition to the anti-PD-1/CD137 mAb combination of cisplatin, a commonly used chemotherapeutic drug for the treatment of ovarian cancer [43], administered at a dose equivalent to those used clinically [38], provided long term remission (most likely cure) in 80% of the treated mice and induced a systemic memory immune response which was antigen specific. The antitumor effect of this combined regimen was confirmed in the murine TC1 lung tumor model with long-term survival 2 of 6 mice receiving combined mAb/cisplatin treatment. The data are concordant with findings from a recent study, which showed that combined administration of anti-PD-1/CD137 mAbs synergized with low-dose radiotherapy to cure established orthotopic AT-3 mouse mammary tumors in a CD8^+^ T cell dependent manner [26]. They also relate to the demonstration that a combination of anti-CD137 mAb with cisplatin induced 60% regression of mouse CT-26 colon carcinoma [38], although we did not observe any beneficial effect against the ID8 tumor unless the anti-CD137 mAb was combined with an anti-PD1 mAb. 

It is noteworthy that both anti-CD137 and anti-PD-1 mAbs, given as single agents and without combination with cisplatin, induced long-term survival of mice which had been transplanted with 3 × 10^6^ ID8 cells 3 rather than 10 days before treatment. Further studies are needed to explore the immune status of treated and control mice with tumors transplanted 3 days before therapy commenced. Further work is also warranted to test whether combined anti-PD-1/CD137 mAbs plus chemotherapy has improved antitumor efficacy in another ovarian cancer model. 

 Blockade of the co-inhibitory receptor PD-1 or its ligand PD-L1 in the clinic using anti-PD-1 or anti-PD-L1 mAb has shown promising results for advanced solid tumors with tolerable immune related adverse events [22,23,44]. Anti-CD137 mAbs have demonstrated toxicity both in some preclinical models [33] and in clinical trials [18,31]. It is noteworthy, therefore, that peritoneal injection of anti-CD137 mAb, a procedure that can be applied clinically in patients with ovarian cancer, did not induce any obvious toxicity such as weight or hair loss either in this study or our preceding experiments in the ID8 model [27]. Our demonstration of additive and possibly synergistic effects combining the anti-PD-1/anti-CD137 mAbs with cisplatin should stimulate further studies to assess the safety and efficacy of similar combinations towards ‘translation’ to the clinic.

## Supporting Information

Figure S1 **Representative dotplots showing the frequency of peritoneal CD3^+^, CD4^+^, CD8^+^ and CD19^+^ lymphocytes and CD44/CD62L expression on peritoneal CD4^+^ and CD8^+^ cells.** Mice (3/group) transplanted i.p. with 3 × 10^6^ ID8 cells 10 days earlier were injected i.p. twice at 4 days interval with control, anti-CD137, anti-CD137 or anti-PD-1/CD137 mAb. Two weeks later, peritoneal lavage from treated mice was analyzed for the frequency of CD3^+^, CD4^+^, CD8^+^ and CD19^+^ cells (upper two panels) and CD44/CD62L expression on peritoneal CD4^+^ and CD8^+^ T cells (bottom two panels) by flow cytometry. Middle panel represents the dotplots for the isotype antibody staining of CD44 and CD62L.
(TIF)

Figure S2 **Representative dotplots showing PD-1 and TIM-3 expression on peritoneal CD4^+^ and CD8^+^ T cells from treated mice.** Mice (3/group) transplanted i.p. with 3 × 10^6^ ID8 cells 10 days earlier were injected i.p. twice at 4 days interval with control, anti-CD137, anti-PD-1 or anti-PD-1/CD137 mAb. Two weeks later, peritoneal CD4^+^ and CD8^+^ T cells from the treated mice was analyzed for the expression of PD-1 and TIM-3 molecules by flow cytometry.
(TIF)

Figure S3 **Cytokine production by peritoneal lavage cells.** Mice (3/group) transplanted i.p. with 3 × 10^6^ ID8 cells 10 days earlier were injected i.p. twice at 4 days interval with control, anti-CD137, anti-PD-1 or anti-PD-1/CD137 mAb. Two weeks later, pooled lavage cells harvested from treated mice were stimulated *in* *vitro* with 50 ng/ml PMA and 1 μg/ml ionomycin for 6 hours prior to the analysis of IL-2 and IFN-γ production in the supernatants by ELISA (R&D systems). The results were analyzed after normalization according to the T cell numbers.
(TIF)
