# Supplementary figures and images for: Combinatorial PD-1 Blockade and CD137 Activation Has Therapeutic Efficacy in Murine Cancer Models and Synergizes with Cisplatin
Source: PLoS One. 2013 Dec 19;8(12):e84927. doi: 10.1371/journal.pone.0084927 (PMC3868659; doi:10.1371/journal.pone.0084927)

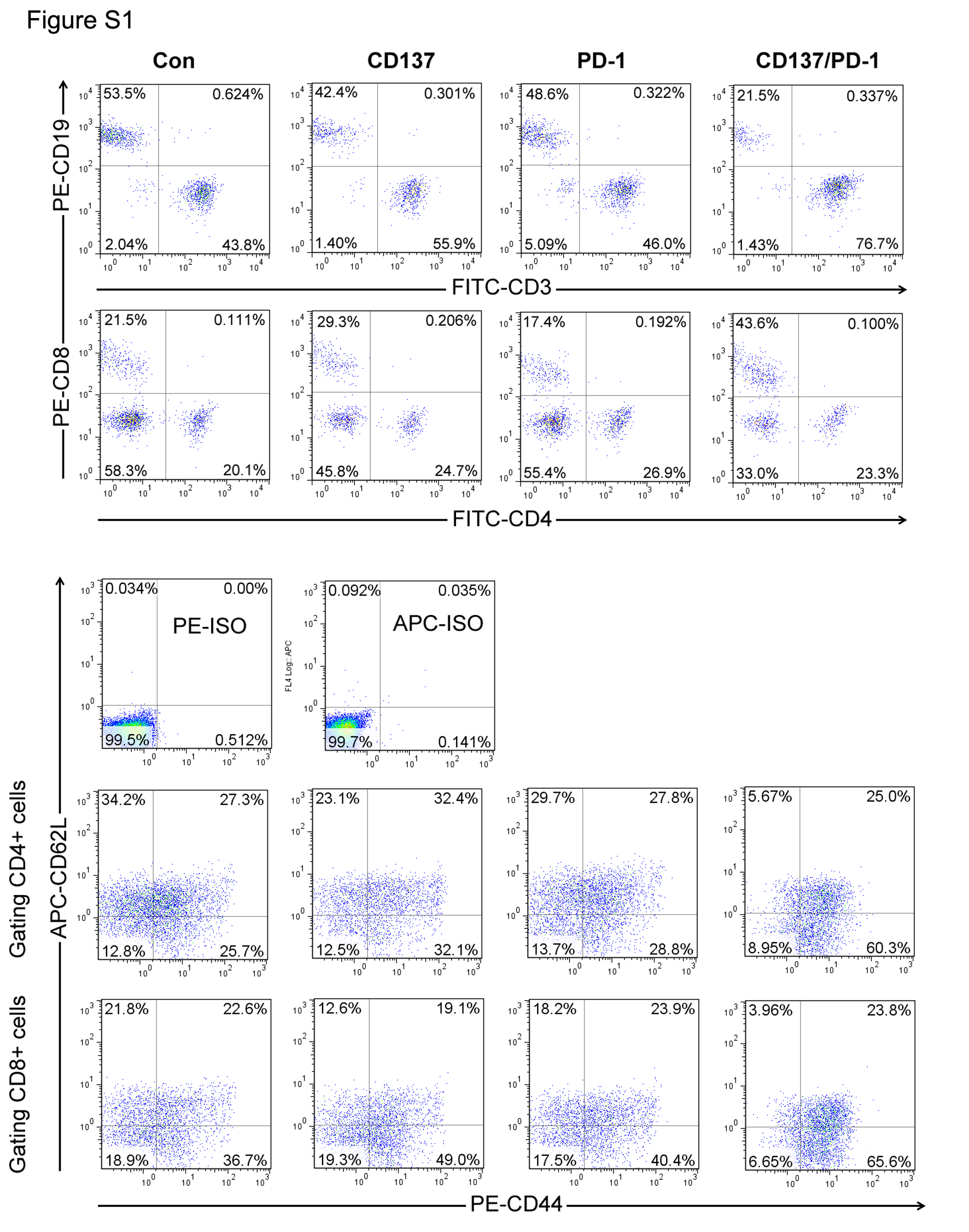

Supplement: Figure S1 — Representative dotplots showing the frequency of peritoneal CD3+, CD4+, CD8+ and CD19+ lymphocytes and CD44/CD62L expression on peritoneal CD4+ and CD8+ cells. Mice (3/group) transplanted i.p. with 3 × 10^6^ ID8 cells 10 days earlier were injected i.p. twice at 4 days interval with control, anti-CD137, anti-CD137 or anti-PD-1/CD137 mAb. Two weeks later, peritoneal lavage from treated mice was analyzed for the frequency of CD3+, CD4+, CD8+ and CD19+ cells (upper two panels) and CD44/CD62L expression on peritoneal CD4+ and CD8+ T cells (bottom two panels) by flow cytometry. Middle panel represents the dotplots for the isotype antibody staining of CD44 and CD62L. (TIF) [file pone.0084927.s001.tif]

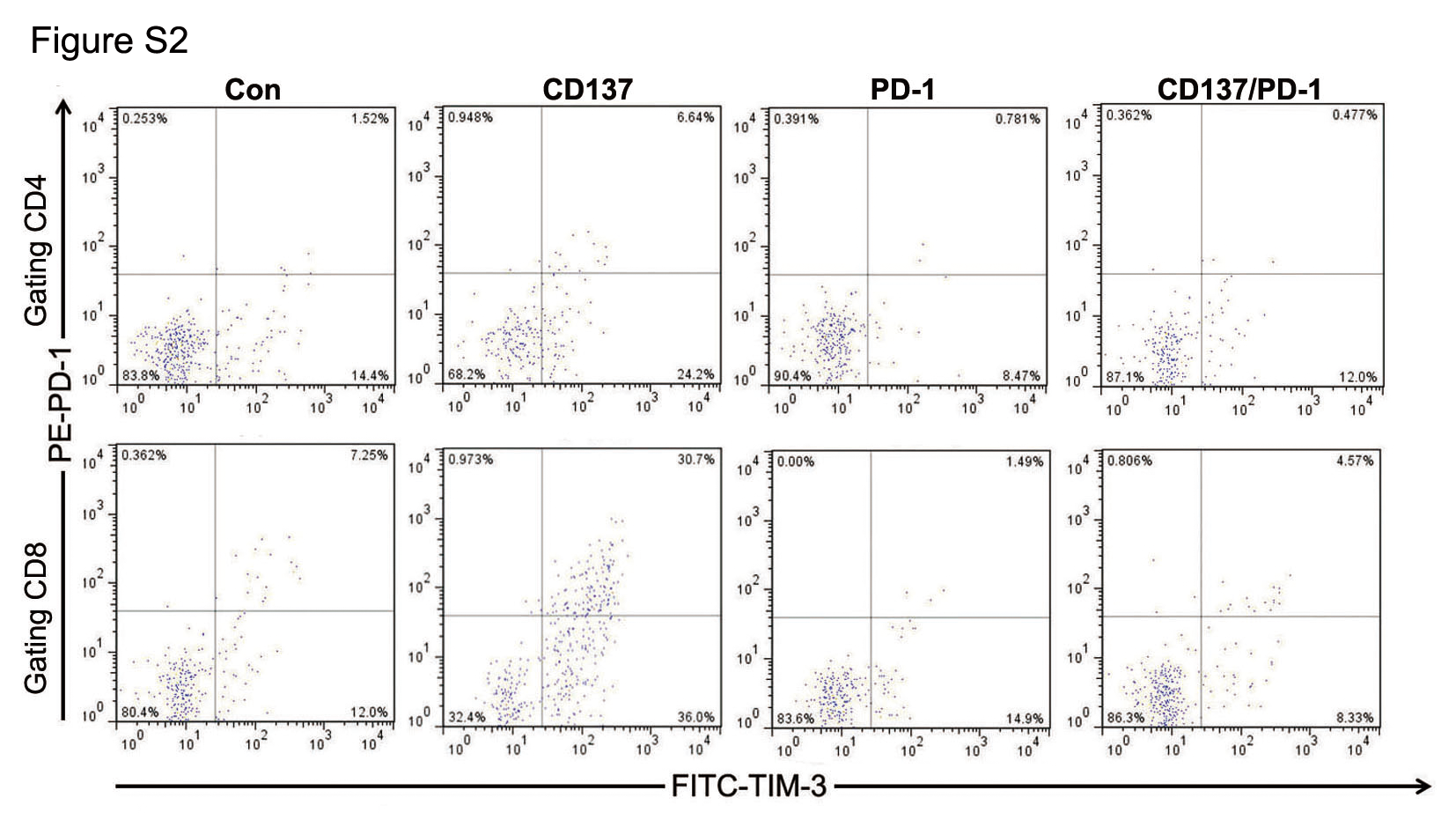

Supplement: Figure S2 — Representative dotplots showing PD-1 and TIM-3 expression on peritoneal CD4+ and CD8+ T cells from treated mice. Mice (3/group) transplanted i.p. with 3 × 10^6^ ID8 cells 10 days earlier were injected i.p. twice at 4 days interval with control, anti-CD137, anti-PD-1 or anti-PD-1/CD137 mAb. Two weeks later, peritoneal CD4+ and CD8+ T cells from the treated mice was analyzed for the expression of PD-1 and TIM-3 molecules by flow cytometry. (TIF) [file pone.0084927.s002.tif]

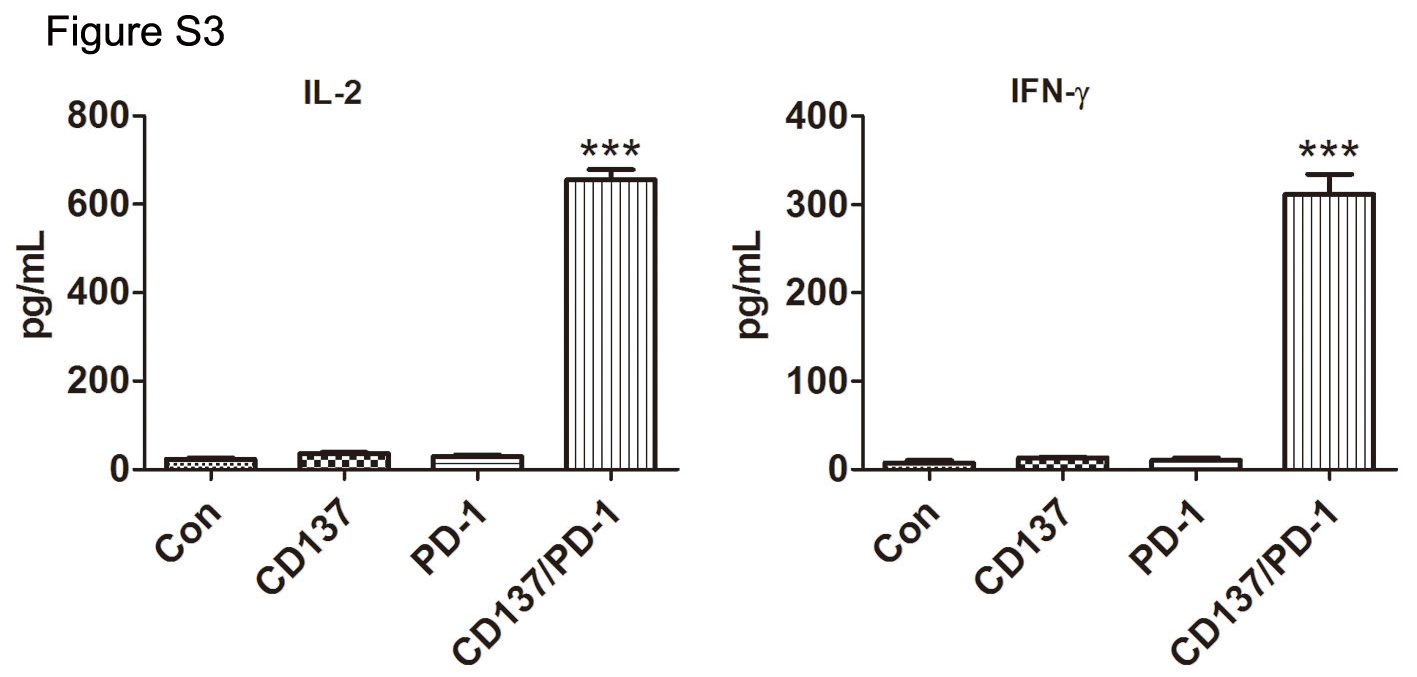

Supplement: Figure S3 — Cytokine production by peritoneal lavage cells. Mice (3/group) transplanted i.p. with 3 × 10^6^ ID8 cells 10 days earlier were injected i.p. twice at 4 days interval with control, anti-CD137, anti-PD-1 or anti-PD-1/CD137 mAb. Two weeks later, pooled lavage cells harvested from treated mice were stimulated in vitro with 50 ng/ml PMA and 1 μg/ml ionomycin for 6 hours prior to the analysis of IL-2 and IFN-γ production in the supernatants by ELISA (R&D systems). The results were analyzed after normalization according to the T cell numbers. (TIF) [file pone.0084927.s003.tif]
